# Association Between Bone Ultrasonometry and Cardiovascular Morbimortality: A Systematic Review and Meta-analysis

**DOI:** 10.1210/jendso/bvaf049

**Published:** 2025-03-18

**Authors:** Clément Vachey, Aurélie Dufour, Pier-Alexandre Tardif, Aboubacar Sidibé, Lynne Moore, Fabrice Mac-Way

**Affiliations:** Department of Medicine, Centre de recherche du CHU de Québec-Université Laval (L’Hôtel-Dieu de Québec Hospital), Nephrology and Endocrinology Axis, Quebec City, QC, Canada G1R 2J6; Faculty and Department of Medicine, Université Laval, Quebec City, QC, Canada G1V 0A6; Department of Medicine, Centre de recherche du CHU de Québec-Université Laval (L’Hôtel-Dieu de Québec Hospital), Nephrology and Endocrinology Axis, Quebec City, QC, Canada G1R 2J6; Faculty and Department of Medicine, Université Laval, Quebec City, QC, Canada G1V 0A6; Population Health and Optimal Health Practices Research Unit, Trauma—Emergency—Critical Care Medicine, Centre de Recherche du CHU de Québec—Université Laval (l'Enfant-Jésus Hospital), Quebec City, QC, Canada G1J 1Z4; Quebec National Institute of Public Health, Quebec City, QC, Canada G1V 5B3; Population Health and Optimal Health Practices Research Unit, Trauma—Emergency—Critical Care Medicine, Centre de Recherche du CHU de Québec—Université Laval (l'Enfant-Jésus Hospital), Quebec City, QC, Canada G1J 1Z4; Department of Social and Preventive Medicine, Université Laval, Quebec City, QC, Canada G1V 0A6; Department of Medicine, Centre de recherche du CHU de Québec-Université Laval (L’Hôtel-Dieu de Québec Hospital), Nephrology and Endocrinology Axis, Quebec City, QC, Canada G1R 2J6; Faculty and Department of Medicine, Université Laval, Quebec City, QC, Canada G1V 0A6

**Keywords:** quantitative ultrasound, bone mineral density, osteoporosis, cardiovascular risk, bone-vascular axis, systematic review

## Abstract

**Context:**

Quantitative ultrasound (QUS) can estimate bone mineral density and predict fracture risk, but its association with cardiovascular outcomes remains unclear.

**Objective:**

We aimed to assess the associations between bone QUS parameters and cardiovascular event risk, cardiovascular mortality (CVM) and all-cause mortality (ACM).

**Data Sources:**

Pubmed, Embase, Cochrane Library databases, and grey literature were searched.

**Study Selection:**

We considered studies including people aged >40 years who reported associations between bone QUS parameters (any bone site) and our outcomes.

**Data Extraction:**

Two reviewers selected eligible studies, extracted and analyzed data, and assessed risk of bias with the Risk of Bias in Non-randomized Studies of Exposure tool. Adjusted hazard ratios (HR) with 95% confidence intervals (CIs), estimated for 1 SD reduction of QUS parameters, were pooled using random effects meta-analyses.

**Data Synthesis:**

We included 9 studies with 275 to 477 683 (median = 3244) participants (follow-up duration range 2.8-12.8 years). All studies presented associations based on calcaneal QUS parameters; only 2 reported associations with cardiovascular events with discordant results. Seven studies reported associations with CVM and 7 with ACM. Meta-analyses based on 3 studies showed that broadband ultrasound attenuation (BUA) was inversely associated with CVM (HR = 1.22, 95% CI: 1.11-1.34, *I*^2^ = 0%) and ACM (HR = 1.16, 95% CI: 1.10-1.23, *I*^2^ = 0%). Meta-analyses, based on 4 and 3 studies, respectively, showed that speed of sound (SOS) was also inversely associated with CVM (HR = 1.19, 95% CI: 1.11-1.27, *I*^2^ = 29%) and ACM (HR = 1.15, 95% CI: 1.07-1.23, *I*^2^ = 0%).

**Conclusion:**

In a cohort of middle-aged individuals, a decrease in calcaneal BUA and SOS were both independently associated with higher cardiovascular and ACM.

Osteoporosis is defined by loss of bone mineral density (BMD) and mass leading to a decreased bone strength and an increased fracture risk [1]. The prevalence of osteoporosis is higher in women and correlates with age. Specifically, it is currently defined by a BMD, measured by dual-energy X-ray absorptiometry (DEXA), as the gold standard, of 2.5 SD or more below the average value for young adults [2, 3]. In the United States, the age-adjusted prevalence of osteoporosis assessed at either the femur neck and/or lumbar spine among adults aged 50 and older was 12.6% in 2017-2018 (19.6% in women and 4.4% in men), a 34% increase over the preceding decade [4].

Many studies have suggested that the presence of osteoporosis is associated with increased cardiovascular morbidity and mortality in men and women [5-9]. Today, cardiovascular diseases (CVDs) are responsible for about 17.9 million deaths annually [10]. Only in the United States, CVDs remain the leading cause of death with an age-adjusted death rate of 224.4 per 100 000 people in 2020 [11]. The relationship between bone dysregulation/osteoporosis and vascular disease has led to the concept of the “bone-vascular axis,” which is described in the general population but is even more important in people with diabetes or chronic kidney disease [12-15]. Even though common risk factors and cross-talk mediators have been suggested [13, 15], the pathophysiological mechanisms between bone disease and vascular pathology still need to be clarified.

Bone assessment by quantitative ultrasound (QUS) has been suggested as an alternative to DEXA for osteoporosis diagnosis and follow-up [16]. Indeed, it is easy to use, portable, and cheaper, and it does not involve ionizing radiation. It also enables measures of bone quality, beyond bone quantity, which is also a determinant of bone strength [17]. The 2 main parameters assessed by QUS are derived from velocity and attenuation of the ultrasound waves through the bone tissue, respectively called speed of sound (SOS) and broadband ultrasound attenuation (BUA). Other parameters can then be mathematically derived such as stiffness index (SI), quantitative ultrasound index, and BMD. Results obtained with QUS cannot classify patients according to bone density cut-off values defined by the World Health Organization [18]. Nevertheless, QUS can detect individuals at high risk for osteoporosis [19], and fracture risk assessment scores based on QUS parameters have been developed [20-22]. Until now, no recommendations regarding antiosteoporotic therapy have been developed based on QUS parameters [23].

While BMD from DEXA has been shown to predict cardiovascular event (CVE) risk or mortality [6, 24, 25], the relationship between QUS and CVD remains unclear [26-28]. Consequently, a systematic review of probing data in the literature combined with a critical analysis could help clarify this issue and potentially support the use of this technique to detect high-risk patients.

The primary objective of this systematic review was to assess the association between bone QUS parameters and cardiovascular events risk in adults aged 40 years and older. Secondary objectives were to (1) assess the association between QUS parameters and cardiovascular mortality and all-cause mortality, (2) identify in which bone site(s) QUS parameters have the strongest correlation with these events, and (3) assess if sex is an effect modifier in these associations.

## Methods

This systematic review was based on the Cochrane systematic review methods and is reported according to the Preferred Reporting Items for Systematic Reviews and Meta-Analysis guidelines [29]. The protocol was registered on the International Prospective Register of Systematic Reviews (PROSPERO CRD42023449623) [30].

### Eligibility Criteria

We used the PECOS approach to define the eligibility criteria of studies (P = population, E = exposition, C = comparison, O = outcomes, S = study design).

#### Population

We considered studies including individuals from the general population aged 40 and older irrespective of their sex, ethnicity, or comorbidities. If a study included some individuals younger than 40, the mean or median age had to be greater than 40 to consider the study eligible.

#### Exposition

We included studies in which QUS were performed to assess BMD with no regard to the bone site (notably calcaneus and radius) or the device used.

#### Comparison

Not applicable.

#### Outcomes

We included studies assessing the following CVEs: myocardial infarction, angina, coronary revascularization procedure, stroke (including transient ischemic attack), lower limb arteriopathy (irrespective of the stage), any aneurysmal disease, mesenteric ischemia, cardiac failure, cardiac arrest, cardiovascular death, and all-cause mortality.

#### Type of studies

We considered observational studies (cohort and case-control) and trials (randomized and nonrandomized). We considered published studies and grey literature. We excluded literature reviews and meta-analyses. Studies under review for publication were not considered.

#### Exclusion criteria

We excluded studies with the following characteristics: studies focusing on individuals younger than 40, in vitro/in vivo studies that used QUS on vessels or organs other than bones, studies assessing surrogate markers of cardiovascular risk such as arterial stiffness or intima-media thickness without any information on the outcomes listed previously, and studies focusing on fracture risk.

### Information Sources and Literature Search

We conducted our search in the following electronic bibliographic databases: PubMed, Embase, and Cochrane (from inception to November 2022, 3rd for Embase, 4th for PubMed, and 17th for Cochrane). We also conducted searches in the grey literature, notably with the Canadian Agency for Drugs and Technologies in Health's Grey Matters online tool [31]. A complementary search was also carried out in the references of eligible studies and in literature reviews. There was no restriction on language, period of publication, or country of origin. The search strategy was developed following an iterative process with the help of an information specialist, first in PubMed (Supplementary Table 1) [32] using keywords and Medical Subject Headings of the main concepts (bone, QUS, CVE, and mortality), and was then adapted to the other databases (Supplementary Table 1) [32].

### Data Collection

#### Study selection

All search results were imported into Endnote 20 (Clarivate Analytics, London, UK), and duplicates were identified and deleted. We developed a study selection form based on our eligibility criteria (Supplementary Table 2) [32]. The study selection process was performed by 2 independent reviewers (C.V. and A.D.). A pilot phase was carried out on a random sample of studies to confirm the agreement of eligibility criteria between the 2 reviewers. Secondarily, all title/abstracts were screened to identify relevant studies. The selected full texts were then assessed according to the eligibility criteria. Discrepancies between the 2 reviewers were discussed, and, in the absence of consensus, a third party (F.M.W.) was consulted to arbitrate.

#### Data extraction

We first developed a data extraction codebook. Then we developed a data extraction form on which the following variables were considered: study characteristics, including name of the first author, the study design, the year of publication, and the country where the study was conducted; population characteristics, including the population size, sex, age, exclusion criteria, and some comorbidities (body mass index, diabetes, smoking); exposure characteristics, including QUS site, parameter measured, and device used; outcome characteristics, including the number of deaths, the types and number of patients experiencing a CVE, the follow-up duration, the statistical model, and the type of association measures [eg, relative risk, hazard ratio (HR), odds ratio], the crude and adjusted association measures, their 95% confidence intervals (CIs), the covariates included in statistical models, and the number of missing data on QUS parameters and vital status. The data collection process was performed in 2 steps by 2 independent reviewers (C.V. and A.D.). First, a pilot phase consisted of extracting the data from 2 studies to check the consistency between the 2 reviewers, and the rest of the extraction was executed in a second phase. Since no discrepancies between the 2 reviewers were observed in the pilot phase, the rest of the extraction was performed without any change to the initial extraction codebook. At the end of the data collection process, any disagreement on the extracted data was discussed by the 2 reviewers, and a third party (F.M.W.) was consulted if there was no consensus between them.

#### Study risk of bias assessment

Two reviewers (C.V. and A.D.) independently assessed the risk of bias in included studies using the Risk of Bias in Non-randomized Studies of Exposure [33]. This tool covers 7 risk of bias domains including bias resulting from confounding, measurement of the exposure, selection of participants, postexposure interventions, missing data, measurement of the outcome, and selection of the reported result [33]. After a successful pilot phase on 2 studies, all included studies were evaluated with the appropriate tool. The 2 reviewers discussed disagreements, and a third party (F.M.W.) was consulted if necessary.

#### Data validation and completion with authors of studies

We contacted authors of 3 included studies to complete extracted data when the information needed was not available and could not be calculated, but we only received 1 response before submission of this manuscript.

### Data Analysis

Meta-analyses were conducted for each parameter-outcome association reported by at least 2 studies with the same type of effect measure using Cochrane RevMan Web (Cochrane Collaboration). Cox models-based effect sizes were estimated as HRs with their 95% CIs, and measures of association based on competing risk models were estimated with subdistribution HRs. The generic inverse variance method was used to pool together the adjusted HRs, and random-effects meta-analyses were performed as fewer than 5 studies were included in each analysis and because we expected that studies’ populations and methods would not be sufficiently homogeneous for fixed effects models. Heterogeneity of the effect estimates between studies was assessed with forest plots and *I^2^* statistics. A 2-sided *P* < .05 was considered statistically significant. Publication bias and asymmetry were assessed through funnel plots and Egger test, respectively. Sensitivity analyses were conducted excluding studies considered at high risk of bias and we further reported associations stratified by sex.

## Results

### Study Selection

**Figure 1.**
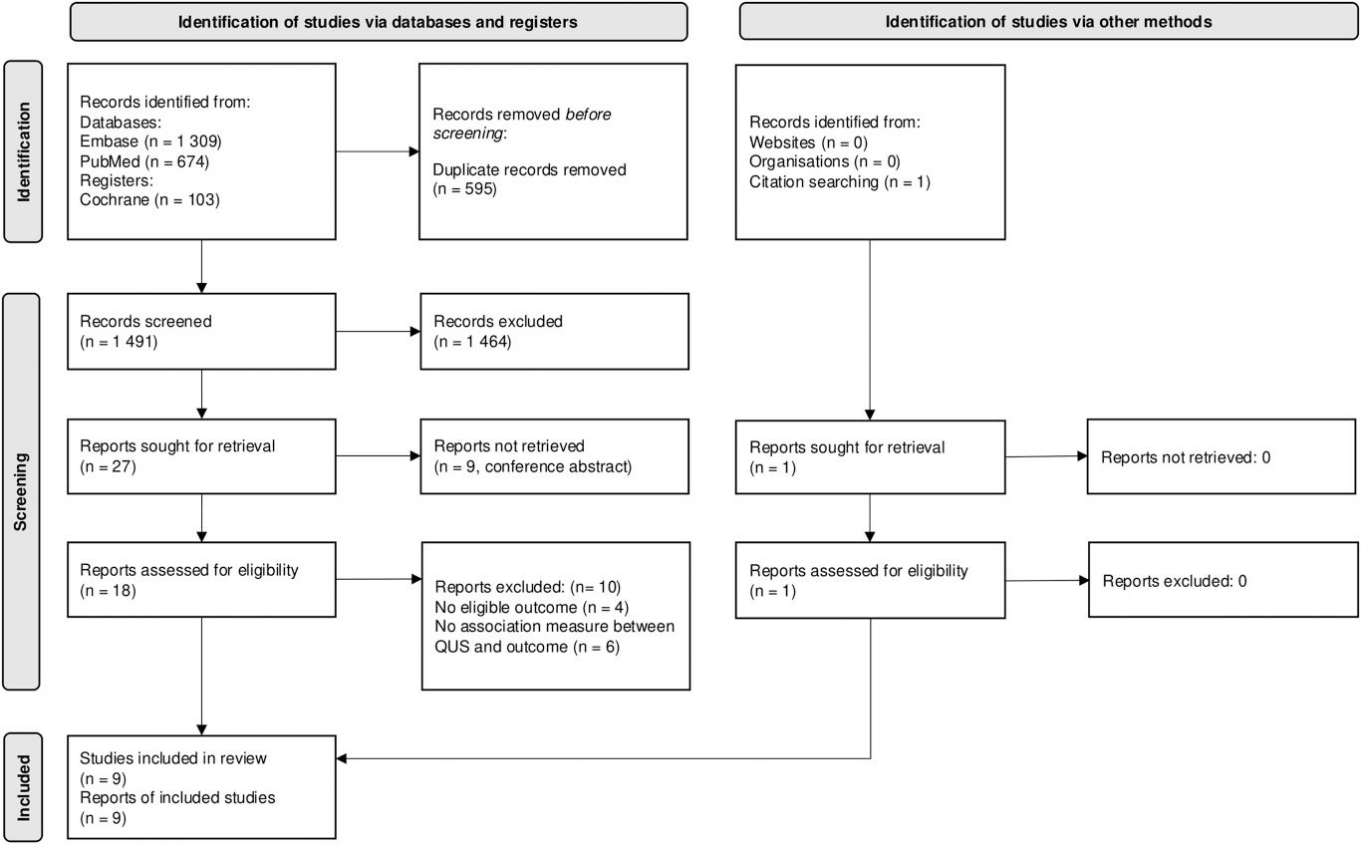
PRISMA flow diagram of the study inclusion process. Adapted from Page et al [29], under the licence CC BY 4.0 (https://creativecommons.org/licenses/by/4.0). Abbreviations: PRISMA, Preferred Reporting Items for Systematic Reviews and Meta-Analysis; QUS, quantitative ultrasound.

The study selection process was described with a Preferred Reporting Items for Systematic Reviews and Meta-Analysis 2020 flowchart (Fig. 1). We identified 2086 potentially relevant studies. After exclusion of 595 duplicates, 1491 titles and abstracts were screened. Of these, 1464 did not meet inclusion criteria, leaving 27 studies. Of these, we excluded 9 studies from conference abstracts, 4 studies because of the absence of an eligible outcome (Tao et al reported an association with Framingham's 10-year CVD risk score [34]; Mizukami et al reported associations with fat mass, muscle mass, and grip strength [35]; Zhen et al reported association with vitamin D level [36]; and Masugata et al reported associations with left ventricular mass, peak early diastolic mitral annular velocity, and hemoglobin level [37]) and 6 studies because of the absence of an association measure reported between a QUS parameter and the defined outcomes [38-43]. Hence, we retained 8 studies [26-28, 44-48]. A search of the grey literature and references from the included articles identified 1 additional article for the review [49]. We therefore included 9 original articles in this systematic review (Fig. 1).

### Characteristics of Included Studies

All included studies were observational, published from 2002 to 2022 (median = 2014), and conducted in high-income countries (Table 1). One study was initially included in a randomized controlled trial of oral calcium supplementation to prevent osteoporotic fractures [28]. Four studies included only women [26, 28, 44, 45], 1 included only men [47], and 4 included both men and women with a majority of women [27, 46, 48, 49]. The number of subjects included ranged from 275 to 477 683 (median = 3244). All individuals were older than 40 with a mean age ranging from 58 to 80.9 (median = 72.1).

**Table 1.** Characteristics of included studies and populations at baseline

References in chronological order	Country	Study design	Number of participants	Age	Women (%)	Follow-up duration (years)	Outcomes	Exclusion criteria (apart from age)
Bauer et al 2002 [44]	United States	Cohort	5816	76.5 (±4.6)*^a^*	5816 (100)	5	All-cause mortality Cardiovascular mortality	Black women, women who were unable to walk without the assistance of another person or who had a bilateral hip replacement [50]
Pinheiro et al 2006 [45]	Brazil	Cohort	275	72.1 (±7.6)*^a^*	275 (100)	5 (maximum)	All-cause mortality Cardiovascular mortality	Endocrinopathy, gastroenteropathy, nephropathy, rheumatic disease, asthma, malnutrition, prolonged immobility (more than 2 months), prolonged corticosteroid therapy, non-Caucasian race
Gonzáles-Macías et al 2009 [26]	Spain	Cohort	5201	72.3 (±5.3)	5201 (100)	2.83	All-cause mortality Cardiovascular mortality	Paget's disease, multiple myeloma, bone metastasis, renal failure (creatinine > 265mcmol/L), hypercalcemia, immobilization for >3 months in the prior year, therapeutic fluoride (>20 mg/day) for more than 3 months during the past 2 years or for more than 2 years at any time in life, a life expectancy <3 years (estimated by the physician), anatomical anomalies of the right foot interfering with ultrasound, participation in another investigation involving drugs
Tsuboi et al 2011 [49]	Japan	Cohort	881	67.7 (±5.3)*^a^*	526 (59.7)	10 (maximum)	All-cause mortality Mortality from cerebro/cardiovascular disease	Individuals who did not have any musculoskeletal checkup during the period considered as baseline
Pfister et al 2014 [46]	United Kingdom	Cohort	13 666	61.5 (±9.0)	7762 (56.8)*^b^*	9.3	Heart failure and death by heart failure	History of heart attack, stroke, or cancer or under medical heart failure treatment
Pye et al 2015 [47]	Italy, Belgium, Sweden, United Kingdom, Spain, Poland, Hungary, Estonia	Cohort	3244	59.8 (±10.8)	0 (0)	4.3 (median)	All-cause mortality Cardiovascular mortality	Subjects unable to provide written, informed consent [51]
Ross et al 2020 [48]	United States	Cohort	738	80.9 (±7.0)	534 (72)	7.3	All-cause mortality	Known dementia
Raisi-Estabragh et al 2020 [27]	United Kingdom	Cohort	477 683	58*^a^* (range 50-67)	263 273 (55)	7 to 12 (range)	Acute myocardial infarction Ischemic heart disease mortality	People unable to consent or complete baseline assessment
Gebre et al 2022 [28]	Australia	Cohort*^c^*	1404	75.2 (±2.7)	1404 (100)	12.8	All-cause mortality Cardiovascular mortality	Women under medication that affects bone mass, life expectancy of less than 5 years, involvement in another clinical trial [52]

^a^This mean (SD) age was not available for the whole cohort but has been estimated with data within different strata presented in the study included or in a previous article for Pinheiro [53].

^b^This number (proportion) was not estimated in the original article and has been estimated with the proportion of men in the different Broadband Ultrasound Attenuation quartiles; Continuous variables are presented as mean (±SD) unless otherwise specified, and categorical variables are presented as number (proportion).

^c^Secondary analysis of data from a randomized trial.

### QUS Parameters

All included studies assessed QUS parameters of the calcaneus. The Sahara bone sonometer (Hologic, Inc, Marlborough, MA) was the most used device (4/9) [26, 27, 47, 48]. In 2 studies, different models of the Achilles sonometer (Lunar, Madison, WI) were used. Bauer et al used the Walker Sonix UBA 575 (Hologic, Waltham, MA), and Pfister et al used the CUBA sonometer (McCue Ultrasonics, Winchester, UK). Different parameters were assessed; BUA and SOS were the most used parameters (2/9 BUA, 2/9 SOS, 2/9 BUA and SOS) [26-28, 44, 46, 47]. SI was used only by Pinheiro et al and Gebre et al. BMD was used by Ross et al (estimated with BUA and SOS by the manufacturer's software) and Tsuboi et al (no explanations on how it was estimated). QUS parameters were considered as a continuous variable in most of the studies except Tsuboi et al, who only considered BMD as a categorical variable (normal vs low if it was less than 80% of the mean in young adults).

### Outcomes

Mean follow-up duration ranged from 2.83 to 12.8 (median = 7.3) years (Table 1). Most of the included studies performed statistical analysis using a Cox model adjusted for potential confounders and reported HRs (7/9) [26, 28, 44-48]. Tsuboi et al used logistic regression to report odds ratios, while Raisi-Estabragh et al used a competing risk model to report subdistribution HRs. Since Tsuboi et al was the only study that did not report time-to-event analysis, it was excluded from the meta-analysis. Two studies reported associations with CVE [27, 46], 7 studies associations with cardiovascular mortality [26-28, 44, 45, 47, 49], and 7 with all-cause mortality [26, 28, 44, 45, 47-49]. Concerning CVEs, Pfister et al used a composite outcome that combined heart failure and death by heart failure. Raisi-Estabragh et al assessed acute myocardial infarction.

### Quality Assessment of Studies

**Figure 2.**
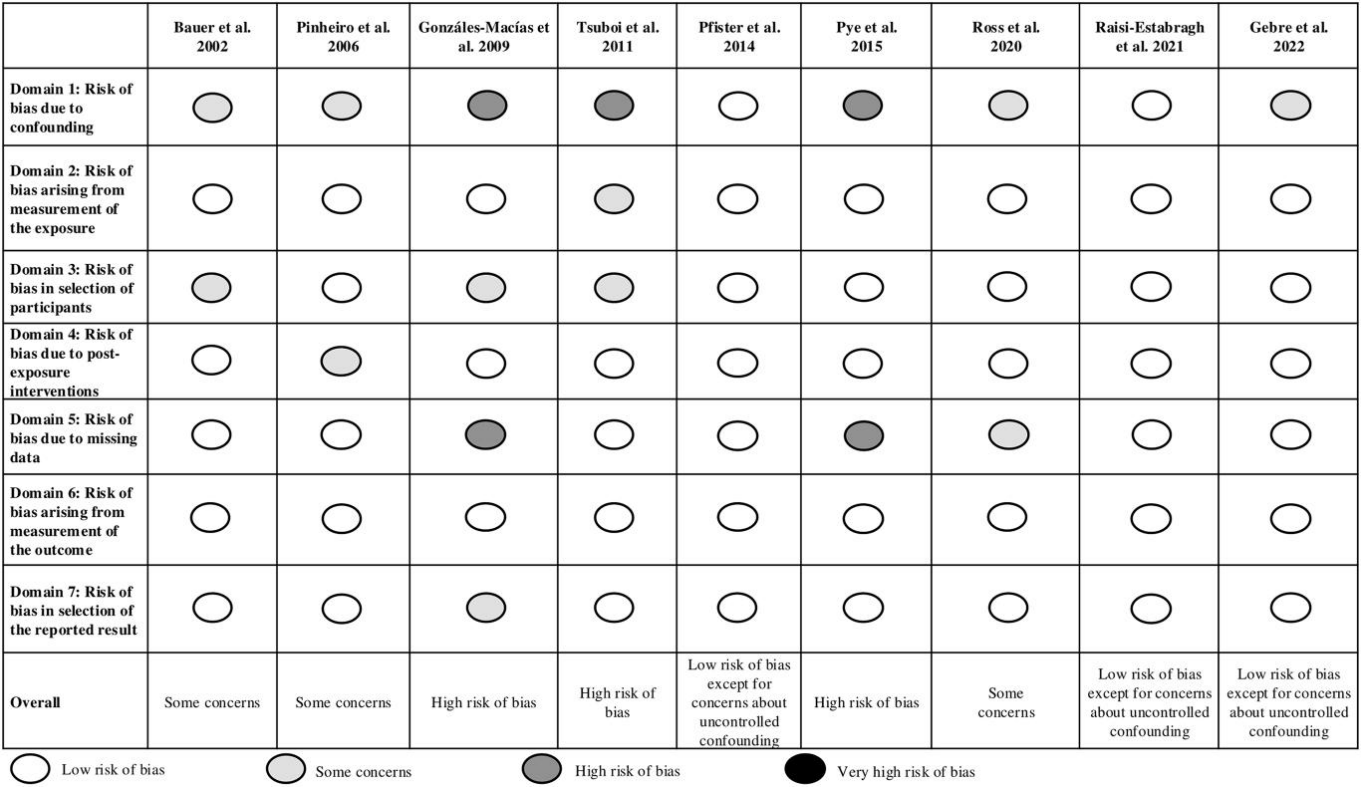
Quality assessment of studies (using Risk of Bias in Non-randomized Studies of Exposure tool).

Three studies were considered at low risk of bias except for concerns about uncontrolled confounding (Fig. 2) [27, 28, 46]. We had some concerns for 3 studies [44, 45, 48], and 3 studies were considered at high risk of bias [26, 47, 49]. Apart from a significant uncontrolled confounding for several studies, risk of bias was notably impacted by selection of participants or missing data (missing data displayed in Supplementary Table 3) [32].

### Associations Between QUS Parameters and Cardiovascular Outcomes and All-cause Mortality

#### CVEs

Only 2 studies reported associations with CVEs (Table 2). Pfister et al observed an inverse correlation between BUA and the HR of CVEs (HR = 1.30 per 1 SD decrease in BUA, 95% CI: 1.12-1.52). Raisi-Estabragh et al only reported sex-stratified associations (see later discussion).

**Table 2.** Association between calcaneal QUS parameters and cardiovascular events (estimated with a Cox model unless otherwise specified)

References in chronological order	QUS parameters	Device	Cardiovascular events	Number of participants with at least 1 event	HR or SHR (95% CI)	Covariates
Pfister et al 2014 [46]	BUA	CUBA Sonometer (McCue Ultrasonics, Winchester, UK)	Heart failure and death by heart failure	256	HR = 1.16 (1.03-1.30)	Age, sex
					HR = 1.30 (1.12-1.52)	Age, sex, BMI, systolic blood pressure, diabetes, cholesterol level, current smoking, alcohol consumption, physical activity, manual occupational social class, low educational level
			Heart failure and death by heart failure in men	170	HR = 1.25 (1.06-1.49)	Age, sex, BMI, systolic blood pressure, diabetes, cholesterol level, current smoking, alcohol consumption, physical activity, manual occupational social class, low educational level
			Heart failure and death by heart failure in women	86	HR = 1.28 (0.93-1.75)	Age, sex, BMI, systolic blood pressure, diabetes, cholesterol level, current smoking, alcohol consumption, physical activity, manual occupational social class, low educational level
Raisi-Estabragh et al 2021 [27]	SOS	Sahara Sonometer (Hologic, Marlboroug, MA)	Acute myocardial infarction in men	5616	SHR = 1.04 (1.01-1.08)*^a^*	Age
					SHR = 1.01 (0.98-1.04)*^a^*	Age, exercise, smoking, deprivation, alcohol
					SHR = 1.01 (0.96-1.07)*^a^*	Age, exercise, smoking, deprivation, alcohol + hypercholesterolemia, diabetes, hypertension
			Acute myocardial infarction in women	2415	SHR = 1.03 (0.99-1.08)*^a^*	Age
					SHR = 0.97 (0.93-1.03)*^a^*	Age, exercise, smoking, deprivation, alcohol
					SHR = 1.00 (0.95-1.05)*^a^*	Age, exercise, smoking, deprivation, alcohol + hypercholesterolemia, diabetes, hypertension

Abbreviations: BMD, bone mineral density; BMI, body mass index; BUA, broadband ultrasound attenuation; CI, confidence interval; HR, hazard ratio; OR, odds ratio; QUS, quantitative ultrasound; SHR, subdistribution hazard ratio; SI, stiffness index; SOS, speed of sound.

^a^Estimated with a competing risk model.

#### Cardiovascular mortality

**Figure 3.**
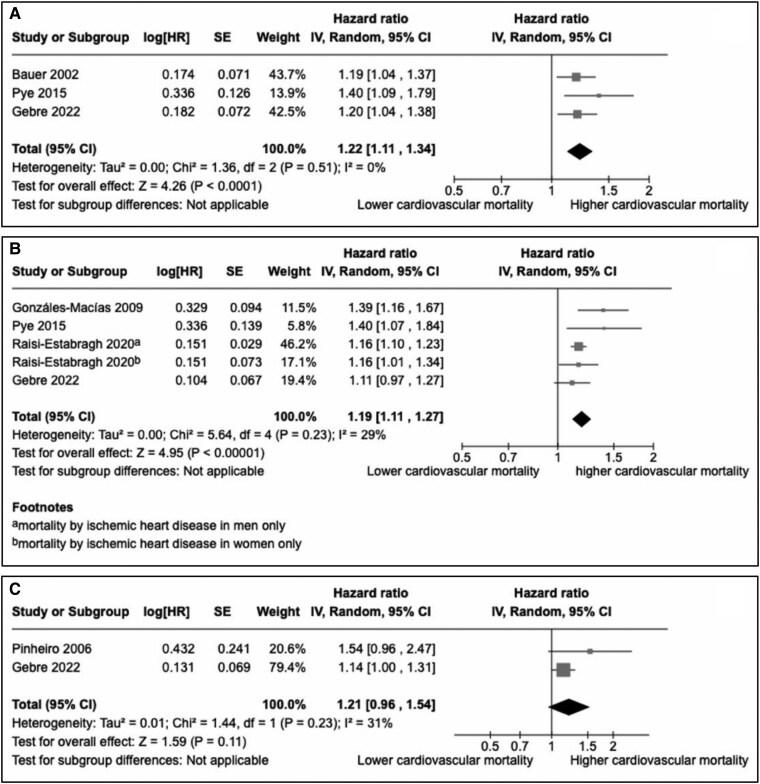
Meta-analyses assessing associations between calcaneal broadband ultrasound attenuation (A), speed of sound (B), stiffness index (C), and cardiovascular mortality (per 1 SD reduction of the quantitative ultrasound parameter). In Raisi-Estabragh, subdistribution HRs were estimated in the study with a competing risk model, considered as HRs for the meta-analysis. Abbreviations: CI, confidence interval; HR, hazard ratio; IV, inverse variance method; Random, random effects.

Seven studies reported associations with cardiovascular mortality [26-28, 44, 45, 47, 49] (Table 3). Most of the results were consistent with an inverse correlation between QUS parameters and cardiovascular mortality (6/7) [26-28, 44, 45, 47]. Three studies were included in the meta-analysis to assess the association between BUA and cardiovascular mortality [28, 44, 47] and 4 studies for the association between SOS and cardiovascular mortality [26-28, 47] (Fig. 3A and 3B); both parameters were associated with a significantly increased risk [respectively, HR = 1.22 (95% CI: 1.11-1.34) and HR = 1.19 (95% CI: 1.11-1.27) per 1 SD reduction], with a low heterogeneity (*I^2^* = 0% and 29%, respectively). Since 2 studies considered at high risk of bias (Gonzáles-Macías et al and Pye et al) were included in meta-analyses, sensitivity analyses excluding these 2 studies have been performed and did not show any significant impact on the results (Supplementary Fig. S1) [32]. In a meta-analysis including 2 studies (Fig. 3C), SI tended to be associated with an increased risk of cardiovascular mortality without reaching statistical significance [HR = 1.21 (95% CI: 0.96-1.54) per 1 SD reduction, *I^2^* = 31%] [28, 45].

**Table 3.** Association between calcaneal QUS parameters and cardiovascular mortality (estimated with a Cox model and expressed per 1 SD reduction of the QUS parameter unless otherwise specified)

References in chronological order	Device	Number of cardiovascular deaths	QUS parameters	HR or SHR or OR (95% CI)	Covariates
Bauer et al 2002 [44]	Walker Sonix UBA 575 (Hologic, Waltham, MA)	250	BUA	HR = 1.19 (1.04-1.37)	Model 1: age, grip-strength, height, weight, health status, smoking, estrogen use, physical activity, history of HTA, diabetes, cardiovascular disease, cancer, stroke
				HR = 1.16 (0.96-1.39)	Model 1 + calcaneal BMD
Pinheiro et al 2006 [45]	Achilles plus (Lunar, Madison, WI)	24	SI	HR = 1.54 (1.09-2.80)	Age, weight, height, BMI, previous fracture, smoking status, comorbidities,*^a^* current physical activity and drugs
Gonzáles-Macías et al 2009 [26]	Sahara (Hologic, Waltham, MA)	42	SOS	HR = 1.39 (1.15-1.66)	Age, thyroxine and hypoglycemic drug treatment, decreased visual acuity
Tsuboi et al 2011 [49]	A-1000 plus II (Lunar, Madison, WI)	39	BMD	low BMD vs normal: OR = 1.23 (0.60-2.54)*^b^*	Age, sex, BMI, lifestyle (smoking, drinking, exercise)
Pye et al 2015 [47]	Sahara (Hologic, Bedford, MA)	70	BUA	HR = 1.4 (1.1-1.8)	Age, center, physical activity, current smoking, comorbidities (none vs 1 or more), general health
			SOS	HR = 1.4 (1.1-1.9)	Age, center, physical activity, current smoking, comorbidities (none vs 1 or more), general health
Raisi-Estabragh et al 2021 [27]	Sahara (Hologic, Marlborough, MA)	IHD deaths in men: 1722	SOS	SHR = 1.16 (1.10-1.23)*^c^*	Age, exercise, smoking, deprivation, alcohol + hypercholesterolemia, diabetes, hypertension
		IHD deaths in women: 388		SHR = 1.16 (1.00-1.33)*^c^*	Age, exercise, smoking, deprivation, alcohol + hypercholesterolemia, diabetes, hypertension
Gebre et al 2022 [28]	Achilles (Lunar, Madison, WI)	223	BUA	HR = 1.20 (1.04-1.38)	Model 1: age, BMI, treatment with calcium + history of smoking, CVD, cancer, diabetes
				HR = 1.28 (1.07-1.53)	Model 1 + hip BMD
			SOS	HR = 1.11 (0.97-1.26)	Model 1: age, BMI, treatment with calcium + history of smoking, CVD, cancer, diabetes
				HR = 1.07 (0.90-1.29)	Model 1 + hip BMD
			SI	HR = 1.14 (1.00-1.31)	Model 1: age, BMI, treatment with calcium + history of smoking, CVD, cancer, diabetes
				HR = 1.16 (0.96-1.39)	Model 1 + hip BMD

Abbreviations: BMD, bone mineral density; BMI, body mass index; BUA, broadband ultrasound attenuation; CI, confidence interval; CVD, cardiovascular disease; HTA, hypertension; IHD, ischemic heart disease; OR, odds ratio; QUS, quantitative ultrasound; SHR, subdistribution hazard ratio; SI, stiffness index; SOS, speed of sound.

^a^Comorbidities included hypertension, diabetes, coronary disease, dyslipidemia, stroke, cancer, pulmonary disease, dementia, falls.

^b^Estimated with a logistic regression, low BMD defined as less than 80% of young adult mean.

^c^Estimated with a competing risk model.

Two studies aimed to determine the independent contribution of QUS parameters and BMD measured by DEXA by entering those 2 variables into statistical models leading to different results [28, 44]. Indeed, Bauer et al did not observe any independent association between BUA and cardiovascular mortality after adjustment with calcaneal BMD (HR = 1.16; 95% CI: 0.93-1.39), whereas Gebre et al did observe a significant association with calcaneal BUA after adjustment with hip BMD (HR = 1.28; 95% CI: 1.07-1.53) but no significant association with SOS and SI.

#### All-cause mortality

**Figure 4.**
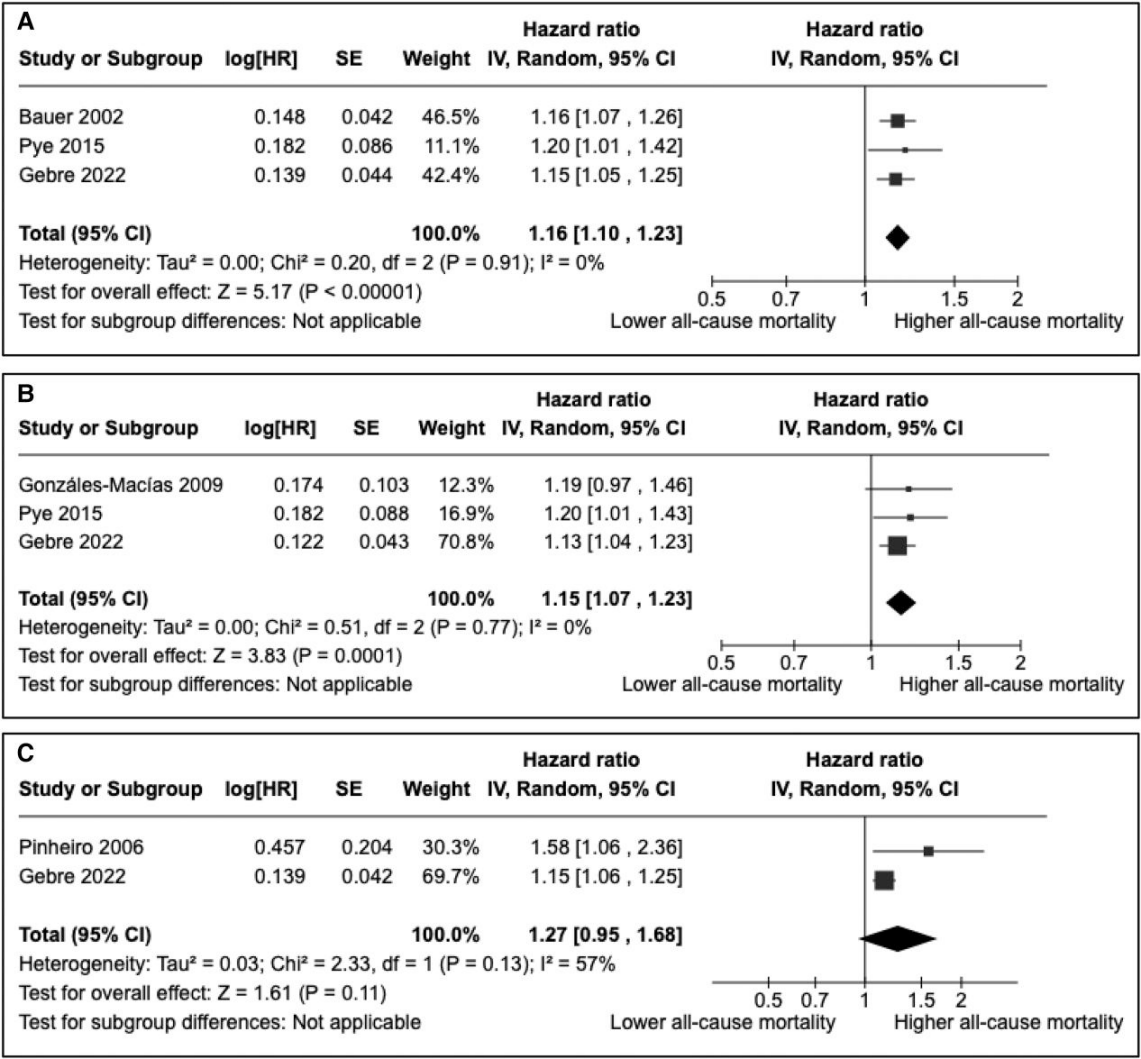
Meta-analyses assessing associations between calcaneal broadband ultrasound attenuation (A), speed of sound (B), stiffness index (C), and all-cause mortality (per 1 SD reduction of the quantitative ultrasound parameter). Abbreviations: CI, confidence interval; HR, hazard ratio; IV, inverse variance method; Random, random effects.

Seven studies reported associations with all-cause mortality [26, 28, 44, 45, 47-49] (Table 4). Similar to cardiovascular mortality, studies also showed an inverse correlation between QUS parameters and all-cause mortality risk. Three studies were included in the meta-analysis to assess the association between BUA and all-cause mortality [28, 44, 47] and 3 studies for the association between SOS and all-cause mortality [26, 28, 47] (Fig. 4A and 4B). Both parameters were associated with a significantly increased risk [respectively, HR = 1.16 (95% CI: 1.10-1.23) and HR = 1.15 (95% CI: 1.07-1.23) per 1 SD reduction], with a low heterogeneity (*I^2^* = 0% for both analyses). Since 2 studies (Gonzáles-Macías et al and Pye et al) considered at high risk of bias were included in meta-analyses, a sensitivity analysis excluding these 2 studies has been performed for BUA and did not show any significant impact on the results (Supplementary Fig. S1) [32]. We could not perform any sensitivity analysis for SOS because an exclusion of these 2 would have left only 1 study. In a meta-analysis of 2 studies (Fig. 4C), SI tended to be associated with an increased risk of all-cause mortality without reaching statistical significance [HR = 1.27 (95% CI: 0.95-1.68) per 1 SD reduction, *I^2^* = 57%] [28, 45].

**Table 4.** Association between calcaneal QUS parameters with all-cause mortality (estimated with a Cox model and expressed per 1 SD reduction of the QUS parameter unless otherwise specified)

References in chronological order	Device	Number of all-cause deaths	QUS parameters	HR or OR (95% CI)	Covariates
Bauer et al 2002 [44]	Walker Sonix UBA 575 (Hologic, Waltham, MA)	677	BUA	HR = 1.16 (1.07-1.26)	Model 1: age, grip strength, height, weight, health status, smoking, estrogen use, physical activity, history of HTA, diabetes, cardiovascular disease, cancer, stroke
				HR = 1.07 (0.96-1.21)	Model 1 + calcaneal BMD
Pinheiro et al 2006 [45]	Achilles plus (Lunar, Madison, WI)	42	SI	HR = 1.58 (1.11-2.47)	Age, weight, height, BMI, previous fracture, smoking status, comorbidities,*^a^* current physical activity and drugs
Gonzáles-Macías et al 2009 [26]	Sahara (Hologic, Waltham, MA)	100	SOS	HR = 1.19 (0,97-1,45)	Age, thyroxine and hypoglycemic drug treatment, chronic obstructive pulmonary disease, decreased visual acuity
Tsuboi et al 2011 [49]	A-1000 plus II (Lunar, Madion, WI)	125	BMD	low BMD vs normal: OR = 2.33 (1.48-3.67)*^b^*	Age, sex, BMI, lifestyle (smoking, drinking, exercise)
Pye et al 2015 [47]	Sahara (Hologic Inc, Bedford, MA)	185	BUA	HR = 1.2 (1.0-1.4)	Age, center, physical activity, current smoking, comorbidities (none vs 1 or more), general health
			SOS	HR = 1.2 (0.99-1.4)	Age, center, physical activity, current smoking, comorbidities (none vs 1 or more), general health
Ross et al 2020 [48]	Sahara (Hologic, Inc, Marlborough, MA)	483	BMD	HR = 1.10 (0.98-1.23)	Age, sex, education, race, BMI, joint pain, musculoskeletal medications, smoking status, global motor function, global cognition, physical activity
Gebre et al 2022 [28]	Achilles (Lunar, Madison, WI)	584	BUA	HR = 1.15 (1.06-1.26)	Model 1: age, BMI, treatment with calcium + history of smoking, CVD, cancer, diabetes
				HR = 1.19 (1.07-1.33)	Model 1 + hip BMD
			SOS	HR = 1.13 (1.04-1.23)	Model 1: age, BMI, treatment with calcium + history of smoking, CVD, cancer, diabetes
				HR = 1.09 (0.98-1.22)	Model 1 + hip BMD
			SI	HR = 1.15 (1.06-1.25)	Model 1: age, BMI, treatment with calcium + history of smoking, CVD, cancer, diabetes
				HR = 1.15 (1.03-1.28)	Model 1 + hip BMD

Abbreviations: BMD, bone mineral density; BMI, body mass index; BUA, broadband ultrasound attenuation; CI, confidence interval; HR, hazard ratio; HTA, hypertension; OR, odds ratio; QUS, quantitative ultrasound; SI, stiffness index; SOS, speed of sound.

^a^Comorbidities included hypertension, diabetes, coronary disease, dyslipidemia, stroke, cancer, pulmonary disease, dementia, falls.

^b^Estimated with a logistic regression, low BMD defined as less than 80% of young adult mean.

#### Effect modification according to sex

Pfister et al observed an inverse correlation between BUA and CVE only for men (HR = 1.25 per 1 SD reduction in BUA, 95% CI: 1.06-1.49). Raisi-Estabragh et al used a competing-risk model and did not observe any significant association between SOS and CVE risk after adjustment for several confounders for men and women. Concerning cardiovascular mortality, Raisi-Estabragh et al was the only study that performed sex-stratified analysis showing less consistent association in women compared to men (ischemic heart disease mortality subdistribution HR of 1.16 per 1 SD reduction in SOS; 95% CI: 1.10-1.23 in men vs 1.16; 95% CI: 1.00-1.33 in women). No study reported sex-stratified associations concerning all-cause mortality.

### Reporting Biases

Two funnel plots were created—1 for the studies included in the meta-analysis for cardiovascular mortality and another for those included in the meta-analysis for all-cause mortality—and are presented in the Supplementary Fig. S2 [32]. Both funnel plots showed signs of asymmetry (Egger test: *P* = .0196 and *P* = .0055, respectively), suggesting that smaller studies with nonsignificant results may have remained unpublished.

## Discussion

Our systematic review aimed to assess the association between bone QUS parameters and CVE risk, cardiovascular mortality, and all-cause mortality. After review, we identified 7 studies reporting associations with cardiovascular mortality and 7 studies reporting an association with all-cause mortality. Meta-analyses showed an inverse correlation between BUA, SOS, and cardiovascular or all-cause mortality risks. The same tendency was observed with SI without reaching statistical significance. We identified only 2 studies that reported associations with CVE while using different QUS parameters and different outcomes, making results difficult to compare.

These results are consistent with previously published data using BMD assessed by DEXA [6, 25], single-photon absorptiometry [5] or dual-photon absorptiometry [9]. Many of these studies have also observed an inverse association between BMD and surrogate markers of cardiovascular risk including vascular calcifications [7, 54] or atherosclerosis [8, 55]. Our results do not demonstrate any potential mechanistic pathways involved in these associations, but they can lead to a few hypotheses. Indeed, Pfister et al observed a significant inverse correlation between BUA and heart failure risk, while Raisi-Estabragh et al did not show any significant association between SOS and acute myocardial infarction risk after adjustment with potential confounders. Although the population included was slightly younger in Raisi-Estrabragh et al (mean age: 58 vs 61.5), this could hardly explain the difference observed. We could hypothesize that the outcome (heart failure and death by heart failure) in Pfister et al might be more associated to QUS parameters than in Raisi-Estabragh's study (myocardial infarction), suggesting that atherothrombotic ischemic disease may not be involved in the association between bone density and cardiovascular morbidity. Instead, valvular or vascular calcifications probably play a prevailing role. This phenomenon has also been noted in subgroups analysis by Pfister et al, who observed that the inverse association was stronger in nonischemic than ischemic heart disease [HR = 1.33 (95% CI: 1.12-1.59) vs HR = 1.22 (95% CI: 0.92-1.61), respectively, per 1 SD reduction in BUA]. Moreover, Raisi-Estabragh et al further reported an inverse relationship between SOS and arterial stiffness, which is consistent with the existing literature [56, 57].

Interestingly, Gebre et al showed that BUA might be associated with cardiovascular and all-cause mortality independently from DEXA-measured hip BMD. This could be explained by the fact that QUS parameters can assess bone characteristics involved in bone strength beyond BMD, like microarchitecture and mechanical properties, bringing potential additional information when evaluating its association with cardiovascular outcomes. However, this association was not reported by Bauer et al. Still, these opposing results may be explained by the use of different bone sites and necessitate additional studies in the future.

Concerning a potential effect modification according to sex, data were too scarce to draw a definitive conclusion. Pfister et al observed a significant association between BUA and heart failure/death by heart failure in men but not in women, and Raisi-Estabragh et al observed an increased risk of mortality by ischemic heart disease in men and not in women. These differences could probably be explained by a lower incidence of cardiovascular morbimortality in women leading to fewer events and reduced statistical power. However, a sex-effect modifier remains highly relevant since both osteoporosis and cardiovascular diseases are widely influenced by sex.

The evidence included in our review has strengths. First, there is a large number of included individuals that come from 4 different continents with very few numbers lost to follow-up. Several QUS parameters and different devices have been used, bringing similar results. Moreover, most authors have taken measures to prevent selection and information bias, and effect measures were adjusted for most relevant confounders in the majority of included studies. Our review has also limitations. First, a conclusion of our results can only be applied for BMD measures at the calcaneus. Recently, Yang and Huang showed an association between DEXA-measured BMD at the femur and cardiovascular disease risk but not with BMD measured at the lumbar spine, arguing for the importance of bone site when studying the relation between bone and vessels [25]. Another limitation comes from the lack of longitudinal evaluation of bone QUS parameters. Indeed, we only have observations that were measured at a single moment. Thus, the measure of the association does not perfectly reflect the processes of bone loss, notably after menopause [58]. Future studies may want to compare the cardiovascular outcomes of individuals with stable BMD vs individuals who experience a fast decrease of their BMD over time. Moreover, because of the observational design of included studies, we cannot exclude the possibility of residual confounding. Hence, we cannot conclude in terms of causal effect in these relationships. Second, concerning the review process, given that only articles mentioning QUS for evaluation of BMD in title or abstract have been included in our search strategy, we may have missed articles that did not specify the method used to measure BMD. This was the case for 1 study (Tsuboi et al), which was later identified in the references from another study. However, we believe that the possibility of missing additional relevant articles from our research strategy is minimal. Finally, we cannot rule out the possibility of reporting bias; as suggested by the funnel plots, the small number of included studies limits the interpretability of this finding. However, we believe that the complementary search conducted in the grey literature may have reduced the impact of such bias.

Our results are in favor of an inverse association between calcaneal QUS parameters and cardiovascular and all-cause mortality. More research is needed to clarify the association with cardiovascular events and to identify effect modifiers and mediators in this relation. Pathophysiological links between bone and vascular metabolisms are currently the object of numerous works. Some common pathogenic factors and cross-talk mechanisms have been identified [12, 13, 15]. Phosphate has notably been described in several studies as a nontraditional risk factor for CVE and all-cause mortality in men [59-61], and many data support an involvement of the fibroblast growth factor-23/Klotho/phosphate axis in the association between BMD and cardiovascular mortality or all-cause mortality [62-64]. A better understanding of this association could support a better screening of osteoporosis in people with cardiovascular disease and better prevention of CVDs in people with osteoporosis. QUS is a cost-effective, harmless, and portable technique that could play a crucial role in the screening of the general population and large-scale clinical research. Moreover, the impact on cardiovascular risk of antiosteoporotic therapies, such as bisphosphonates, still needs to be determined. A better understanding of the cardiovascular effect of these therapies and an identification of people who could have significant benefits from these drugs could lead to an expansion of the indications of treatment, including indications with a cardiovascular purpose only, independent of bone metabolism.

In conclusion, studies suggest that calcaneal QUS parameters are associated with cardiovascular morbidity and mortality. Hence, a low calcaneal mass may potentially be viewed as a marker of cardiovascular risk. The impact of the evolution of QUS parameters over time and the difference between men and women still need to be evaluated.

## Data Availability

Some datasets generated and analyzed during the current study are not publicly available but are available from the corresponding author on reasonable request.

## Registration

This systematic review is registered as PROSPERO CRD42023449623. No protocol has been published. We chose to amend the registered protocol by performing a meta-analysis since the number of included studies reporting associations for the same parameter and bone site was sufficient.
